# Supplementary figures and images for: Native protein delivery into rice callus using ionic complexes of protein and cell-penetrating peptides
Source: PLoS One. 2019 Jul 30;14(7):e0214033. doi: 10.1371/journal.pone.0214033 (PMC6667096; doi:10.1371/journal.pone.0214033)

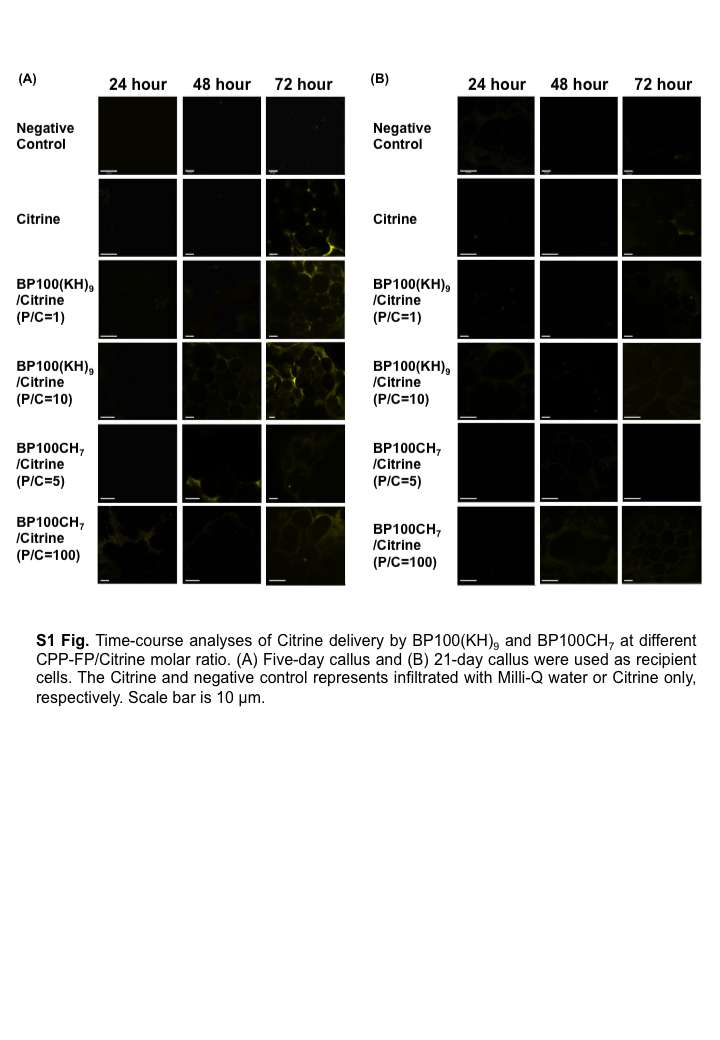

Supplement: S1 Fig — (A) Five-day callus and (B) 21-day callus were used as recipient cells. The Citrine and negative control represents infiltrated with Milli-Q water or Citrine only, respectively. Scale bar is 10 μm. (TIFF) [file pone.0214033.s001.tiff]

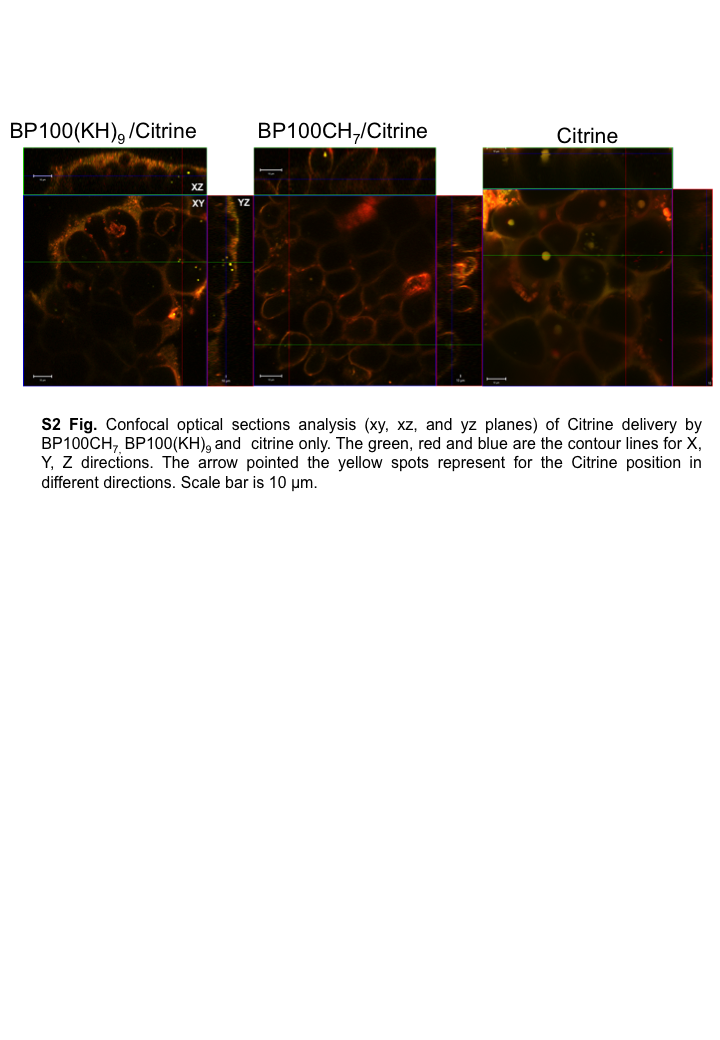

Supplement: S2 Fig — The green, red and blue are the contour lines for X, Y, Z directions. The arrow pointed the yellow spots represent for the Citrine position in different directions. Scale bar is 10 μm. (TIFF) [file pone.0214033.s002.tiff]

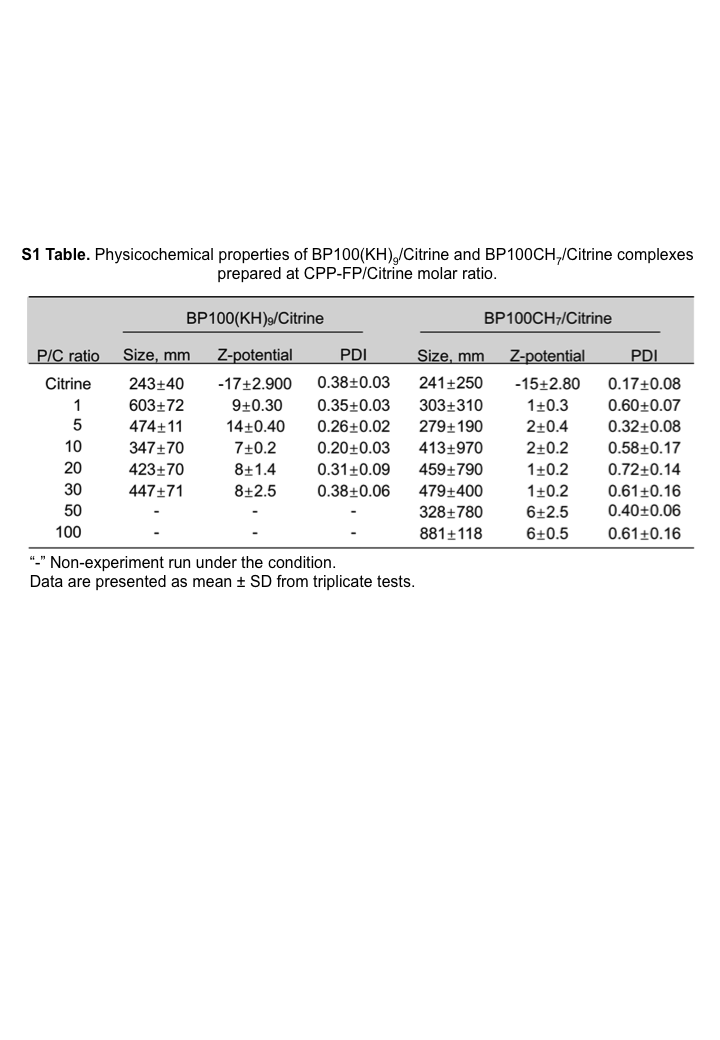

Supplement: S1 Table — (TIFF) [file pone.0214033.s003.tiff]
